# Economic Value of Fosaprepitant-Containing Regimen in the Prevention of Chemotherapy-Induced Nausea and Vomiting in China: Cost-Effectiveness and Budget Impact Analysis

**DOI:** 10.3389/fpubh.2022.913129

**Published:** 2022-07-12

**Authors:** Xinglu Xu, Yuwen Bao, Kai Xu, Zhuolin Zhang, Ningli Zhao, Xin Li

**Affiliations:** ^1^Department of Regulatory Science and Pharmacoeconomics, School of Pharmacy, Nanjing Medical University, Nanjing, China; ^2^Department of Health Policy, School of Health Policy and Management, Nanjing Medical University, Nanjing, China; ^3^Center for Global Health, School of Public Health, Nanjing Medical University, Nanjing, China

**Keywords:** cost-effectiveness, budget impact analysis, antiemetic, chemotherapy-induced nausea and vomiting, fosaprepitant

## Abstract

**Objective:**

The purpose of this study was to evaluate the cost-effectiveness and budget impact of fosaprepitant (FosAPR)-containing regimen for the prevention of chemotherapy-induced nausea and vomiting (CINV) among patients receiving high emetogenic chemotherapy (HEC) from the Chinese payer's perspective.

**Methods:**

A decision tree model was established to measure the 5-day costs and health outcomes between the APR-containing regimen (aprepitant, granisetron, and dexamethasone) and FosAPR-containing regimen (fosaprepitant, granisetron, and dexamethasone). Clinical data were derived from a randomized, double-blind controlled trial on Chinese inpatients who received HEC. Quality-adjusted life-years (QALYs) were used to estimate the utility outcomes and the incremental cost-effectiveness ratio (ICER) was calculated to assess the economics of FosAPR. A static budget impact model was developed to assess the impact of FosAPR as a new addition to the National Reimbursement Drug List (NRDL) on the medical insurance fund within 3 years in Nanjing, China.

**Results:**

Compared with APR, FosAPR had a mean health-care savings of ¥121.56 but got a reduction of 0.0001815 QALY, resulting in an ICER of ¥669926.19 per QALY. Deterministic sensitivity analysis revealed that the cost of APR was the most influential factor to the ICER. The cost of FosAPR and the complete control rate of the delayed period also had a high impact on the results. According to the probabilistic analysis, the acceptability of FosAPR was more than 80% when the Chinese willingness-to-pay (WTP) was ¥215,999. FosAPR would lead to a 3-year medical insurance payment increase of ¥1.84 million compared with ¥1.49 million before FosAPR entered NRDL in Nanjing. The total budget increased with a cumulative cost of ¥694,829 and covered an additional 341 patients who benefited from FosAPR in Nanjing. Deterministic sensitivity analysis showed that the model of budget impact analysis was stable.

**Conclusion:**

FosAPR had a similar treatment effect to APR but was cost-effective in China at the current WTP threshold. The total budget of medical insurance payments of Nanjing slightly increased year by year after the inclusion of FosAPR. Its inclusion in the NRDL would be acceptable and also expand the coverage of patients who benefited from FosAPR.

## Introduction

Cancer is now a global concern and a heavy burden on the health systems of all countries in the world. According to the “Global Cancer Statistics” published by the American Cancer Society, there were 18.1 million new cases of cancer worldwide and 9.6 million cases of cancer deaths in 2018 (1). While in 2020, the number of new cancer cases rose to 19.3 million and almost 10.0 million cancer deaths around the world (2). In recent years, new treatment methods for malignant tumors, such as immunotherapy, targeted therapy, and genetic therapy, have developed rapidly. However, as one of the commonly used treatments for cancer, chemotherapy is still one of the most effective methods. Drugs of chemotherapy are generally cytotoxic, most of which lack targeting for tumor cells. They may also harm the normal cells of the body, leading to more adverse drug reactions. Digestive system reaction, alopecia, bone marrow suppression, liver, and kidney function damage are common toxic and side effects of chemotherapy. Meanwhile, some cytotoxic drugs have specific side effects. For example, doxorubicin has cardiotoxicity, which may cause damage to myocardial cells, and even lead to heart failure in severe cases. Different chemotherapy strategies had different adverse drug reactions (ADR).

Of all the adverse reactions, chemotherapy-induced nausea and vomiting (CINV) is one of the most common side effects of chemotherapy. It makes patients suffer from low-quality lives and has a large negative impact on patient compliance. Also, it can decline patients' performance status and even make them withdraw from the chemotherapy (3–5). A clinical study by Zhang and Li (6) showed a low rate of antiemetic guideline compliance in chemotherapy patients, implying that medical care still had a lot of shortcomings in this field. Based on the risk of emetic and percentage of incidence of vomiting, it was widely accepted that we divided anti-tumor drugs into four grades: (1) high, with over 90% risk of vomiting; (2) moderate, with 30–90% risks; (3) low, with 10–30% risks; and (4) minimal, with below 10% risks of vomiting (7). The risk here is the incidence of vomiting without preventive treatment. Generally speaking, the emetic of platinum-based chemotherapy regimens is considered at moderate and high grades. In light of the time of occurrence, CINV can be divided into three phases, acute phase (0–24 h), delayed phase (25 h), and anticipatory CINV. Anticipatory CINV occurred more frequently in patients who experienced CINV in previous chemotherapy. Previous literature indicated that the incidence of emesis in the delayed phase was correlated with, but not dependent on that in the acute phase (8). In addition, there are many patient-related risk factors for CINV. Systematic reviews and guidelines identified that history of nausea or vomiting, female sex, younger age, and expectancy of CINV could all influence or even increase the incidence of CINV in patients (9–13). As a result, it is important to improve the quality of care for the patients who received high-emetic chemotherapy (HEC) carefully from various aspects.

One of the effective preventions of nausea and vomiting is to give prior antiemetic drugs before and during chemotherapy. 5-hydroxytryptamine (5-HT_3_), substance P (SP), dopamine, acetylcholine, and histamine are neurotransmitters closely related to CINV. In recent years, 5-HT_3_ was considered to play an important role in preventing CINV, especially in the acute phase. Furthermore, substance P is a regulatory polypeptide that can bind to neurokinin (NK) receptors and emerges as the dominant driver of the CINV in the delayed phase. The anti-inflammatory effects of glucocorticoids are also used clinically to prevent the occurrence of delayed CINV. Therefore, 5-HT_3_ receptor antagonists (5-HT_3_ RA), NK-1 receptor antagonists (NK-1 RA), glucocorticoids, general antiemetic drugs proton-pump receptor inhibitors (PPI), and H_2_ receptor antagonists are several types of drugs, which are conventionally used to prevent nausea and vomiting. Ondansetron, granisetron, palonosetron, dexamethasone, aprepitant (APR), etc. are all commonly used antiemetic drugs. For moderate or high risks of vomiting, a co-prescription of two or three antiemetic medications would be frequently given as the guideline-recommended.

Aprepitant is a type of NK-1 RA, it can selectively inhibit the link between the substance P and NK-1 receptors, thus blocking the pathway to vomiting. Some clinical trials and observational studies revealed that aprepitant could statistically significantly improve the prevention of emesis compared to the control regimen and this effect was also observed in children (8, 14–16), which showed its outstanding antiemetic function. However, to completely control the CINV, aprepitant needs to be used 48 h after the chemotherapy is dosed, accompanied by much inconvenience. As a result, fosaprepitant (FosAPR) was synthesized to solve the inexpediency of aprepitant. Fosaprepitant is a prodrug of aprepitant, which can convert into aprepitant after absorption. Its bioavailability is almost 100% which is much higher than aprepitant. Secondly, fosaprepitant is easy to use and has the characteristics of a quick effect. Its intravenous infusion can be completed in 30 min before the start of chemotherapy and can rapidly converse to active compounds in the liver (17).

Even though appropriate antiemetic precautions could stop ~70–80% of CINV episodes (18), the status quo of the CINV is not optimistic. There are more than 30% of patients still suffering from nausea and vomiting after receiving antiemetic treatment (4). Therefore, rescue treatments are badly required when CINV occurs. The basic principle of rescue treatments is to recheck the antiemetic regimens and give different types of antiemetic drugs as appropriate. As for the rescue drugs, except for 5-HT_3_ RA, guidelines and expert consensus recommended treatments including promethazine, metoclopramide, olanzapine, lorazepam, haloperidol, scopolamine, omeprazole, etc.

Since 2007, studies have been conducted in seven countries to evaluate the economic value of prophylactic antiemetic regimens (19–24). Most of them showed favorable results with APR. For instance, in 2019, Kashiwa et al. demonstrated that the economic efficiency of the addition of FosAPR to prophylactic antiemetic therapy for outpatient HEC was not cost-effective, although the addition of APR was cost-effective in the context of the Japanese healthcare system (25). However, due to huge differences in different healthcare systems, the economic value of a pharmaceutical product may vary by country. A study in the United States found that APR had little cost-effectiveness benefit.

Based on the Institute for Clinical and Economic Review (ICER) Value Framework 2.0, the economic efficiency and affordability of health technology should be simultaneously considered for inclusion in the reimbursement list (26). Moreover, whether a new drug can be afforded by public health insurance funds is the key issue for its value evidence. Since 2019, enterprises must submit the economic evidence from Cost-effectiveness Analysis (CEA) and Budget Impact Analysis (BIA) to the National Healthcare Security Administration (NHSA) for inclusion in the National Reimbursement Drug List (NRDL) of China.

However, the comprehensive economic assessment of FosAPR-containing regimen therapy for cancer patients in China remains unknown. Especially, there is a lack of comparison between FosAPR and APR. This study aimed to evaluate the cost-effectiveness and budget impact of the FosAPR-containing regimen from the perspective of Chinese payers for patients who received HEC in the context of the Chinese healthcare setting. The BIA of FosAPR was crucial to provide modeling estimates to support evidence-based decision-making for drug reimbursement. It could also be used for budget or resource planning to ensure that the medical insurance funding was affordable if FosAPR would be included in the NRDL.

## Methods

### Cost-Effectiveness Analysis

#### Overview

**Figure 1:**
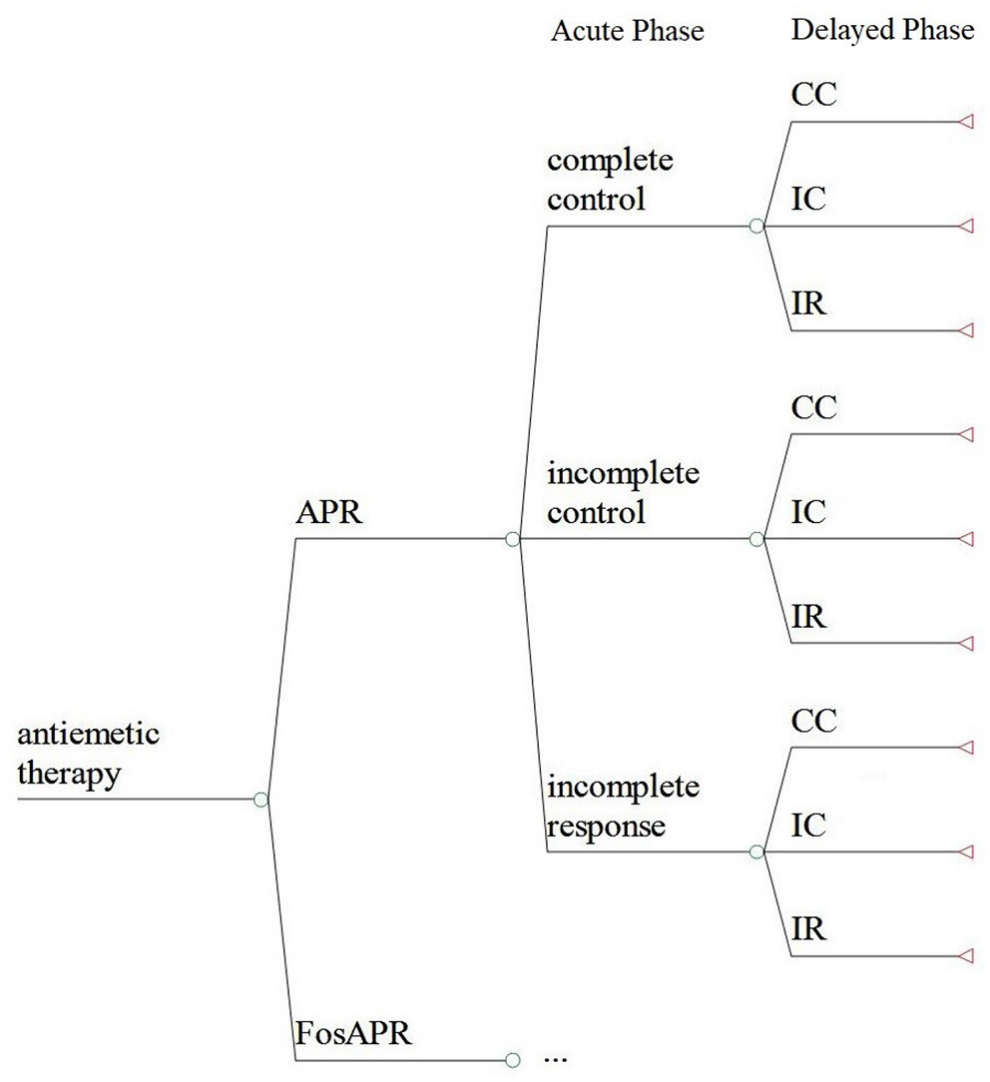
The decision model of the cost-effectiveness analysis. FosAPR, fosaprepitant; APR, aprepitant; CC, complete control; IC, incomplete control; IR, incomplete response.

A decision model which has three stages and two phases was established to describe the therapy process (Figure 1). Complete control (CC), incomplete control (IC), and incomplete response (IR) were set to represent three clinical outcomes the patients went through in the acute phase (0–24 h) and delayed phase (25–120 h). CC means no nausea, no vomiting, and no rescue therapy, IC means some emesis but no use of rescue therapy, and IR means nausea and vomiting while getting some use of rescue therapy. The covering time of research was 5 days including the administration of chemotherapy and the preventive antiemetic drug. Microsoft Office Excel 2007 was used to conduct the analysis.

#### Clinical Data

The clinical data were stemmed from a randomized, double-blind phase 3 clinical trial, a multicentre study that compared the safety and efficacy of FosAPR with APR in Chinese cancer patients (27). Cancer patients to be enrolled were required to be between 18 and 75 years old receiving high-risk emetic chemotherapy and had a good physical condition, with an Eastern Cooperative Oncology Group (ECOG) performance status score between 0 and 2. In addition, the patient's expected survival time should have been longer than 3 months. A total of 645 patients in 21 centers in China who received chemotherapy including high-risk emetogenic drugs were included in this trial. In this study, patients were divided into APR (*n* = 317) or FosAPR (*n* = 328) group randomly. There was no significant difference in demographic data between the two groups. The baseline characteristics of the patients in this study are shown in Table 1. Because the clinical data was selected from the published report of clinical trial, ethical approval was not required for the study on participants in accordance with the local legislation and institutional requirements.

**Table 1:** The baseline characteristics of patients in the study.

	**FosAPR group (*n* = 328)**	**APR group (*n* = 317)**
Age, median (range)	55 (20–79)	53 (18–74)
**Sex (%)**		
Male	163(49.70%)	163(51.42%)
Female	165(50.30%)	154(48.58%)
**ECOG (%)**		
0	64(19.57%)	64(20.19%)
1	251(76.76%)	238(75.08%)
2	12(3.67%)	15(4.73%)
Cisplatin-contained (%)	263(80.2%)	234(73.8%)

*ECOG, Eastern Cooperative Oncology Group performance status score. One data missing in the FosAPR group*.

A triple therapy regimen (FosAPR (150 mg IV d1) or APR (125 mg orally d1; 80 mg orally d2–d3) plus granisetron and dexamethasone) was used to prevent vomiting. Olanzapine tablets (5 mg orally once) and metoclopramide injection (10 mg IM once) were used as rescue drugs in the research. The complete response rate (CRR) and complete control rate (CCR) in both phases were elicited from the trial report (Table 2). The incidences of rescue treatment were 6.40 and 2.84% in FosAPR group and APR group, respectively (27).

**Table 2:** Health state probabilities of the clinical trial.

**Response (%)**	**FosAPR regimen (*****n*** **= 328)**		**APR regimen (*****n*** **= 317)**	
	**Acute phase**	**Delayed phase**	**Acute phase**	**Delayed phase**
Complete control	84.45	72.56	87.38	74.45
Incomplete control	11.28	18.60	8.52	18.93
Incomplete response	4.27	8.84	4.10	6.62

*FosAPR: fosaprepitant; APR: aprepitant*.

#### Costs and Utility

Costs were estimated from the perspective of the Chinese healthcare system and presented in CNY. Only direct medical costs are incorporated in this study, including drug therapy, rescue therapy, hospitalization, and drug administration. Antiemetic treatment drugs and rescue drugs were described above. The costs of hospitalization consisted of blood routine, blood biochemistry, prescription preparation, and basic consumables. Due to the chemotherapy, all patients were the inpatients, so the hospitalization costs of the two groups were equal in this research. The unit prices of drugs and medical examinations were derived from the surveys that were conducted in local hospitals (e.g., The Affiliated Bayi Hospital of Nanjing University of Chinese Medicine and Nanjing Drum Tower Hospital). The report of the WHO Macroeconomic Committee recommends 1–3 times gross domestic product (GDP) per capita as the threshold for judging the cost-effectiveness of drug. Triple Chinese 2020 GDP per capita (¥215,999) was set to be the willingness-to-pay (WTP) threshold. However, the impact of adverse drug reactions (ADR) was not considered in this study. Also, the discount rate was not considered in the simulation as the trial only lasted for 5 days.

The utility value of three health states of CINV in the Chinese population has not been reported in a clinical trial or published literature before. Referring to the previous studies (25, 28, 29), we separately set the three utility values as 0.9, 0.7, and 0.3. The costs and utility values are summarized in Table 3.

**Table 3:** Costs and utility values in the study.

**Type of value**	**Value**	**Range**	**Distribution**	**References**
Cost (¥)				
Drugs in FosAPR	512.48	±25%	Gamma	Local charge
Drugs in APR	634.88	±25%	Gamma	Local charge
Inpatient	469.34	–	Gamma	Local charge
Rescue therapy	23.61	–	Gamma	Local charge
Utility				
CC	0.9	±0.1	Beta	(23, 26, 27)
IC	0.7	±0.1	Beta	(23, 26, 27)
IR	0.3	±0.1	Beta	(23, 26, 27)

*FosAPR: fosaprepitant; APR: aprepitant; CC: complete control; IC: incomplete control; IR: incomplete response*.

Health outcomes were measured by quality-adjusted life years (QALYs). The sum of the 5-day QALYs was calculated as follows:


$$\begin{array}{l} 5\text{-day QALY}=\left({\text{U}}_{\text{Acute}}\;*\;1\text{ day}+{\text{U}}_{\text{Delayed}}\;*\;4\text{ days}\right)/365\text{ days} \end{array}$$

The 5-day QALYs of the acute and delayed phases are presented in Table 4. To evaluate the cost-effectiveness of FosAPR compared with APR, the incremental cost-effectiveness ratio (ICER) was calculated and was used to compare with the WTP threshold. If the ICER value is less than the WTP threshold, it means that FosAPR is cost-effective compared to APR. Otherwise, it is not economical.


$$\begin{array}{l} ICER=\left(Cost_{\text{FosAPR}}-Cost_{\text{APR}}\right)/\left(QALY_{\text{FosAPR}}-QALY_{\text{APR}}\right) \end{array}$$

**Table 4:** Utility values of outcomes in acute and delayed phases.

**Health state in the acute phase (0–24 h)**	**Health state in the delayed phase (25–120 h)**	**Base-case 5-day QALY**
CC	CC	0.0123
	IC	0.0101
	IR	0.0047
IC	CC	0.0118
	IC	0.0096
	IR	0.0041
IR	CC	0.0104
	IC	0.0082
	IR	0.0027

*QALY, quality-adjusted life year; CC, complete control; IC, incomplete control; IR, incomplete response*.

#### Sensitivity Analysis

For testing the uncertainty and robustness of the model, we conducted deterministic sensitivity analysis and probabilistic sensitivity analysis (PSA). CCR of FosAPR and APR in acute and delayed phases, cost of antiemetic drugs, utilities of three health states, and incidences of rescue were considered as the influencing factors of the outcomes. The range of CCR in the sensitivity analysis was 95% confidence interval (CI), while the fluctuation of costs and incidences of rescue was set to be ±25%. The range of utility value was plus or minus 0.1 of its baseline value. Tornado diagram was drawn to show the sensitivity of the influencing factors. In PSA, the distributions of cost and utility were gamma and beta distribution, respectively. CCR, CRR, and incidences of rescue were all in normal distribution. Microsoft Office Excel 2007 was used to perform a Monte-Carlo simulation of 1,000 samples and a scatter plot was made to demonstrate the variation in data. The cost-effectiveness acceptability curve was also generated according to the results of the simulation. The curve illustrated the probability of FosAPR being cost-effective at different WTP thresholds.

### Budget Impact Analysis

#### Overview

According to the practical principles of BIA published by the International Society for Pharmacoeconomics and Outcomes Research (ISPOR) and relevant guidelines, a static budget impact model was developed to assess the impact of inclusion of FosAPR in the NRDL on the health insurance budget of Nanjing in 2022–2024 (30, 31). Microsoft Excel 2007 was used to build the BIA model of FosAPR. Deterministic sensitivity analysis was performed on the base-case BIA results to test the uncertainty of model parameters.

#### Target Population

The target population was tumor patients treated with HEC regimens in Nanjing, China. The total population was elicited from the Nanjing Statistical Yearbook 2020 released by the Nanjing Municipal Bureau of Statistics (32). At the end of 2019, the total population of Nanjing was 8.5 million. The incidence of cancer in China is about 201 per 100,000 people (33). Due to the lack of relevant epidemiological data in China, it was not possible to calculate the total number of patients receiving HEC. Consulting the research of Restelli et al. (34) in Italy, patients who suffered from lung cancer, head and neck cancer, gastric cancer, testicular cancer, and bladder cancer were selected as the population of HEC. By calculation, there were about 5,793 cancer patients receiving HEC regimens in Nanjing. The morbidities of five cancers are shown in Table 5.

**Table 5:** Morbidities of five cancers.

**Tumor types**	**Morbidity**	**Reference**
Lung cancer	36.09/100,000	(35)
Head and neck cancer	4.32/100,000	(36)
Gastric cancer	21.98/100,000	(35)
Testicular cancer	0.3/100,000	(37)
Bladder cancer	5.46/100,000	(35)

#### Market Share

In China, 5-HT3 receptor antagonists occupied the majority of the antiemetic drugs market. The market share of antiemetics for CINV in 2021 was obtained from Jiangsu Institute of Medicine Information, which is a large database with medicine procurement records covering 35 secondary and tertiary hospitals and 27 primary health institutions in Nanjing, China. Nanjing is the capital of Jiangsu Province, which is located in the southeast coast of China, with a population of over 9 million (in 2020) residing across 11 municipal districts. The total gross domestic product (GDP) of this city was 1,481.8 billion CNY (~US$228.8 billion) in 2020, which represents the middle-and-upper level of economic development in Eastern China. Due to its superior geographical position, the 62 sampled healthcare institutions in Nanjing usually provide medical services to the residents of 3 provinces and 1 municipality (Jiangsu Province, Zhejiang Province, Anhui Province, and Shanghai Municipality) in China. Therefore, the data from the drug market in Nanjing was regional representative. The market share of antiemetics for CINV in Nanjing in 2021 was still dominated by 5-HT3 receptor antagonists, with dolasetron, tropisetron, ondansetron, palonosetron, and azasetron as the top five. The market share was 32.03, 24.75, 16.43, 14.60, and 6.85%, followed by Granisetron and APR, which accounted for 4.96 and 0.39%, respectively. The brand-name APR was produced by Merck and was approved for import registration in July 2013 in China. But the brand name FosAPR was not listed. In October 2019, generic FosAPR injections produced by Chia Tai Tianqing Pharmaceutical Co., Ltd., and Hausen Pharmaceutical Co., Ltd. were successively approved for marketing in China. Therefore, we set the market share of this drug to 0% in 2021. Ramosetron and netupitant and palonosetron capsules were not considered for the BIA for they have not been included in the local health insurance drug list of Nanjing. Based on market research, database, and literature (38), FosAPR would compete with 5-HT3 receptor antagonists and APR in the antiemetic drug market of CINV after being included in the NRDL. There was no difference in the ratio of market share decline of all seven competing products. The market share of each 5-HT3 receptor antagonist and NK-1 RA (APR) was set to decline by a linear ratio of 2% every year (39). For example, the market share of Dorasetron in 2021 is 24.75%. After a linear decrease of 2%, the market share is expected to be 24.75%^*^ (1–0.02) = 24.25% in 2022 and 24.75%^*^ (1–0.02) ^∧^2 = 23.77% in 2023. The changes of the market share of each drug within 3 years after the entry of FosAPR into the health insurance drug list are shown in Table 6.

**Table 6:** Market share changes of antiemetics within 3 years.

**Year**	**Dorasetron**	**Tropisetron**	**Ondansetron**	**Palonosetron**	**Azasetron**	**Granisetron**	**APR**	**FosAPR**
2021	24.75%	14.60%	32.03%	16.43%	6.85%	4.96%	0.39%	0.00%
2022	24.25%	14.31%	31.39%	16.10%	6.71%	4.86%	0.38%	2.00%
2023	23.77%	14.02%	30.76%	15.78%	6.58%	4.76%	0.37%	3.96%
2024	23.29%	13.74%	30.14%	15.46%	6.45%	4.67%	0.36%	5.88%

*APR, aprepitant; FosAPR, fosaprepitant*.

#### Costs of Antiemetic Therapies

Only drug costs were incorporated into the budget. The cost of consumables and administration such as intravenous injection could be ignored compared with drug costs. Generally, all patients with HEC regimens need to be hospitalized for drug delivery, so the cost of hospitalization and adjuvant drugs were consistent. To simplify the results and facilitate comparison, these costs were not included in the analysis. The unit prices of drugs were originated from the survey of the drug purchase prices in Nanjing. The Chinese Society of Clinical Oncology (CSCO) Guidelines for Prevention and Treatment of Nausea and Vomiting Caused by Anti-tumor Therapies 2019 was referred to make the dosing plan (40). The administration schemes and the prices of a single course of therapies are shown in Table 7.

**Table 7:** Daily administration schemes and prices of a single course for prevention of CINV.

**Drugs**	**Strength**	**Unit price (¥)**	**Dosing**	**Total cost**
Dorasetron	12.5 mg/injection	144.50	12.5 mg, day1, IV	144.50
Tropisetron	5 mg/injection	37.72	5 mg, day1, IV	37.72
Ondansetron	8 mg/injection	33.99	8–16 mg, day1, IV	67.98
Palonosetron	0.25 mg/injection	53.80	0.25 mg, day1, IV	53.80
	0.5 mg/tablet	154.40	0.5 mg, day1, PO	154.40
Azasetron	10 mg/injection	39.70	10 mg, day1, IV	39.70
Granisetron	3 mg/injection	7.90	3 mg, day1, IV	7.90
	1 mg/tablet	10.58	2 mg, day1, PO	21.16
APR	125 mg,80 mg/tablet	191.67	125 mg, day1; 80 mg, day2-3, PO	575.00
FosAPR	150 mg/injection	450.00	150 mg, IV, day1	450.00
	150 mg/injection	458.00	150 mg, IV, day1	458.00

*IV, intravenous injection; PO, oral; APR, aprepitant; FosAPR, fosaprepitant*.

Palonosetron, granisetron, and FosAPR were available in two specifications, so the average cost per course was used. FosAPR and APR must be used in combination with palonosetron (0.25 mg, day 1) and dexamethasone tablets (6 mg, day 1; 3.75 mg, days 2–3). 5-HT3 receptor antagonists should be combined with dexamethasone injection (10 mg, per day 1). The cost of dexamethasone was too low to take into account. The treatment cycle was calculated to be about 6 cycles by the weighted average of the first-line chemotherapy regimens for the cancers included in the study (41–47). Accordingly, the study assumed that antiemetic drugs were administered 6 times within a year. Based on the individual out-of-pocket (OOP) standard of medical insurance in Nanjing (48), the OOP ratio of all kinds of antiemetic drugs varied from 0.1 to 0.5. The proportion of reimbursement was about 80% defined by the Nanjing Medical Insurance Bureau. Take ondansetron as an example, its OOP ratio in Nanjing for urban employee was 0.1 and its annual cost was ¥407.88. The cost covered by medical insurance would be 407.88^*^(1–0.1) ^*^80% = 293.67 CNY. Assume that FosAPR was covered by health insurance, with an OOP ratio of 0.5 and an estimated 80% of reimbursement similar to APR. The final single course cost, annual cost, and cost covered by health insurance of each antiemetic drug are summarized in Table 8.

**Table 8:** Single course cost, annual cost, and cost covered by medical insurance of antiemetic drugs.

**Drugs**	**Single course cost**	**Annual cost**	**Cost covered by medical insurance**
Dorasetron	144.50	867	346.80
Tropisetron	37.72	226.32	162.95
Ondansetron	67.98	407.88	293.67
Palonosetron	104.10	624.6	199.87
Azasetron	39.70	238.2	171.50
Granisetron	14.53	87.18	62.77
APR	628.80	3,772.8	1,560.77
FosAPR	507.80	3,046.8	1,270.37

*APR, aprepitant; FosAPR, fosaprepitant*.

#### Research Perspective

The proportions of reimbursement were not uniform in China, and different proportions of reimbursement may have a different effect on the results of BIA. The BIA was based on the health insurance reimbursement policy in the urban area of Nanjing. All the patients in the study were assumed to be urban employees in Nanjing. To facilitate the calculation and the comparison, only the medical costs of antiemetic drugs were included, and the cost of adjunctive drugs and hospitalization were excluded.

## Results

### Cost-Effectiveness Analysis

#### Base-Case Analysis

The total costs of FosAPR and APR regimens were ¥983.33 and 1,104.89, respectively. While the two regimens obtained a benefit of 0.0110993 and 0.0112807 QALY. Compared with APR, FosAPR had a mean health-care savings of ¥121.56, but APR exceeded 0.0001815 QALY to FosAPR, resulting in ICER of ¥669,926.19 per QALY (Table 9). Although the cost-effectiveness ratios of the two antiemetic regimens were both smaller than the WTP threshold, the ICER was much higher than the WTP threshold, which meant that FosAPR was cost-effective compared with APR in the context of the Chinese healthcare system.

**Table 9:** Base-case results of FosAPR and APR in CEA.

	**Cost (¥)**	**QALY**	**CER**	**ICER**
FosAPR	983.33	0.0110993	88,594.19	–
APR	1,104.89	0.0112807	97,945.06	–
Incremental	−121.56	−0.0001815		669,926.19

*FosAPR, fosaprepitant; APR, aprepitant; QALY, quality-adjusted life year; ICER, incremental cost-effectiveness ratio*.

#### Sensitivity Analysis

**Figure 2:**
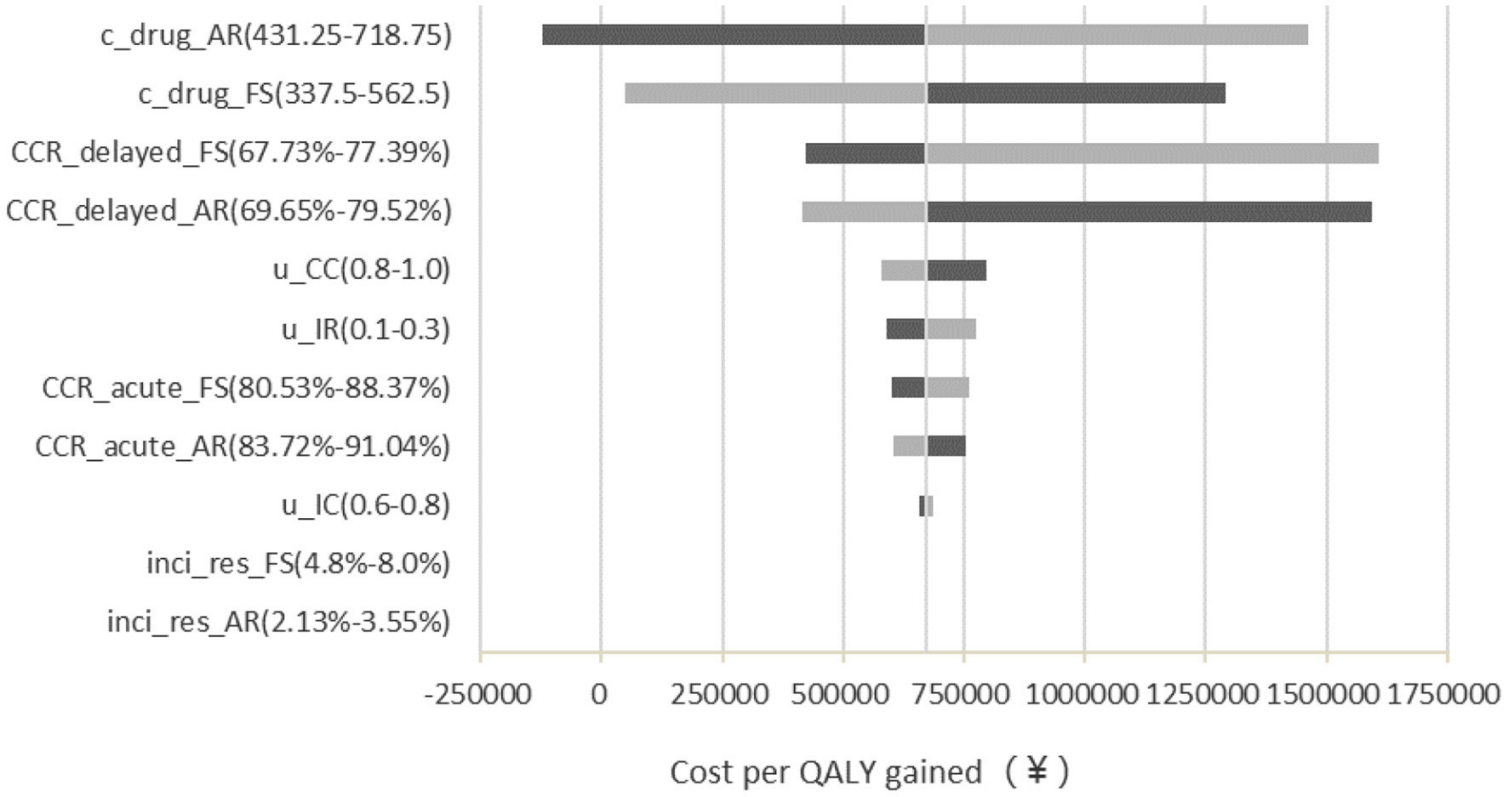
Deterministic sensitivity analysis tornado diagram of FosAPR vs. APR in CEA. c, cost; CCR, complete control ratio; u, utility; FS, fosaprepitant; AR, aprepitant; inci_res, incidence of rescue therapy.

One-way sensitivity analysis results demonstrated that the costs of two antiemetic drugs were the most influential factors in the outcomes, especially the cost of APR (Figure 2). If the price of APR is reduced more than ¥85 or FosAPR increases its price, then the APR group might be cost-effective. Otherwise, FosAPR was a more recommended option when choosing antiemetic drugs before performing high-emetic chemotherapy.

**Figure 3:**
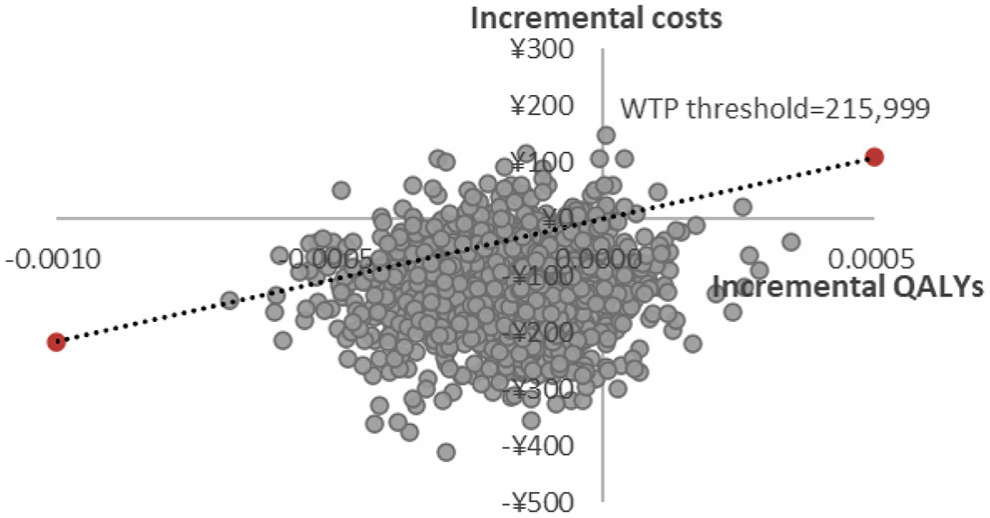
Probabilistic sensitivity analysis of FosAPR vs. APR. WTP, willingness-to-pay; QALY, quality-adjusted life year.

**Figure 4:**
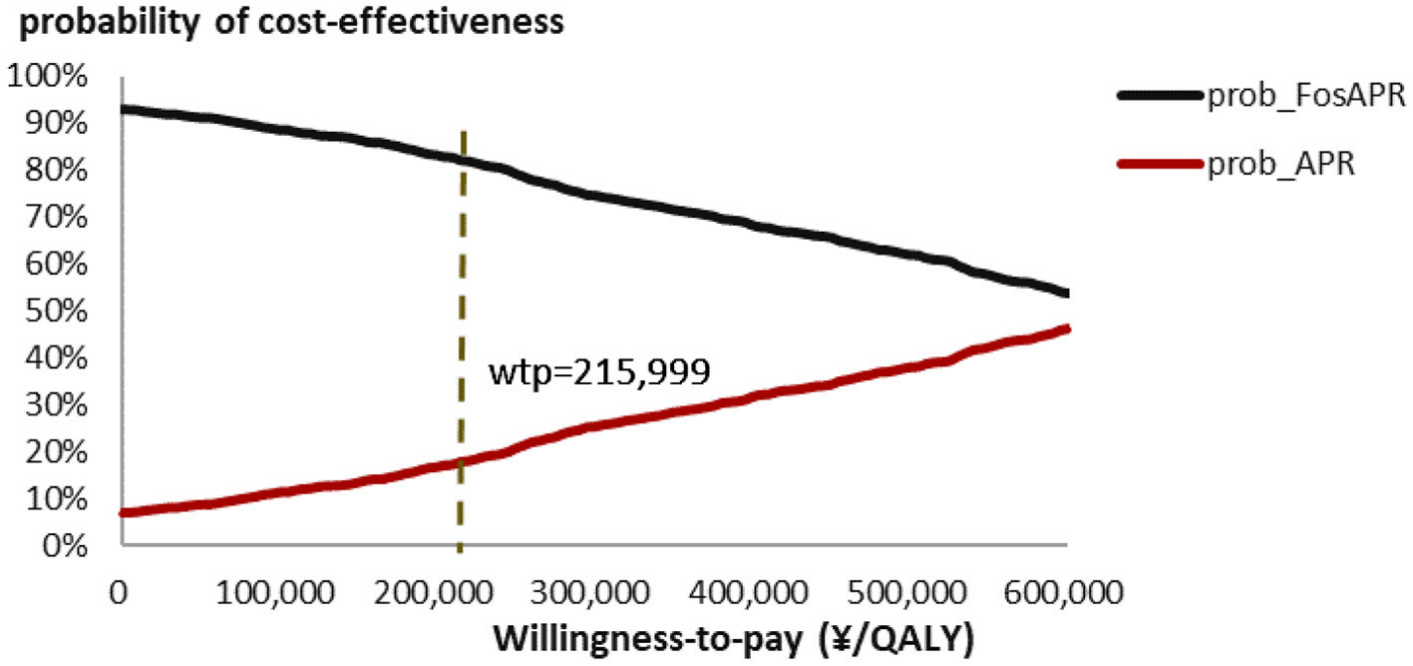
Cost-effectiveness acceptability curve of FosAPR. WTP, willingness-to-pay; QALY, quality-adjusted life year; prob, probability of cost-effectiveness.

PSA scatter plot showed that most scatter were in the third quadrant, which meant FosAPR was more economical compared with APR. At the same time, more than two-thirds of the scatters were below the WTP threshold line, implying that people's acceptance of FosAPR was far higher than that of APR at the current WTP threshold (Figure 3). As well as the acceptance curve displayed, the probability that FosAPR could be cost-effective was over 80% when the WTP threshold was ¥215,999. As the WTP threshold rose, the probability of cost-effectiveness gradually decreased (Figure 4). The curve implied that the higher the threshold, the higher the tendency of patients to choose APR. The outcomes of PSA both prompted that FosAPR was economical in China.

### Budget Impact Analysis

#### Base-Case Analysis

After the inclusion of FosAPR in the NRDL, it is expected that the target population in Nanjing using FosAPR will increase by 116, 229, and 341 in 2022–2024, respectively, and the total medicare payments will increase from ¥1.49 million in 2021 to ¥1.84 million in 2024, indicating a certain rise in the amount of medical insurance payment. The incremental BI of 3 years would be 117,361, 232,376 and 345,090, respectively, accounting for 0.073, 0.135, and 0.188 of the total expenditure of that year. The cumulative total cost of the medical insurance payments in 3 years reached ¥694,828 (Table 10).

**Table 10:** Total medical insurance payments of FosAPR before and after inclusion (CNY).

**Year**	**Pre-inclusion**	**Post-inclusion**	**Difference**	**Cumulation**
2022	1,491,153	1,608,514	117,361	117,361
2023	1,491,153	1,723,529	232,376	349,738
2024	1,491,153	1,836,243	345,090	694,828

#### Sensitivity Analysis

**Figure 5:**
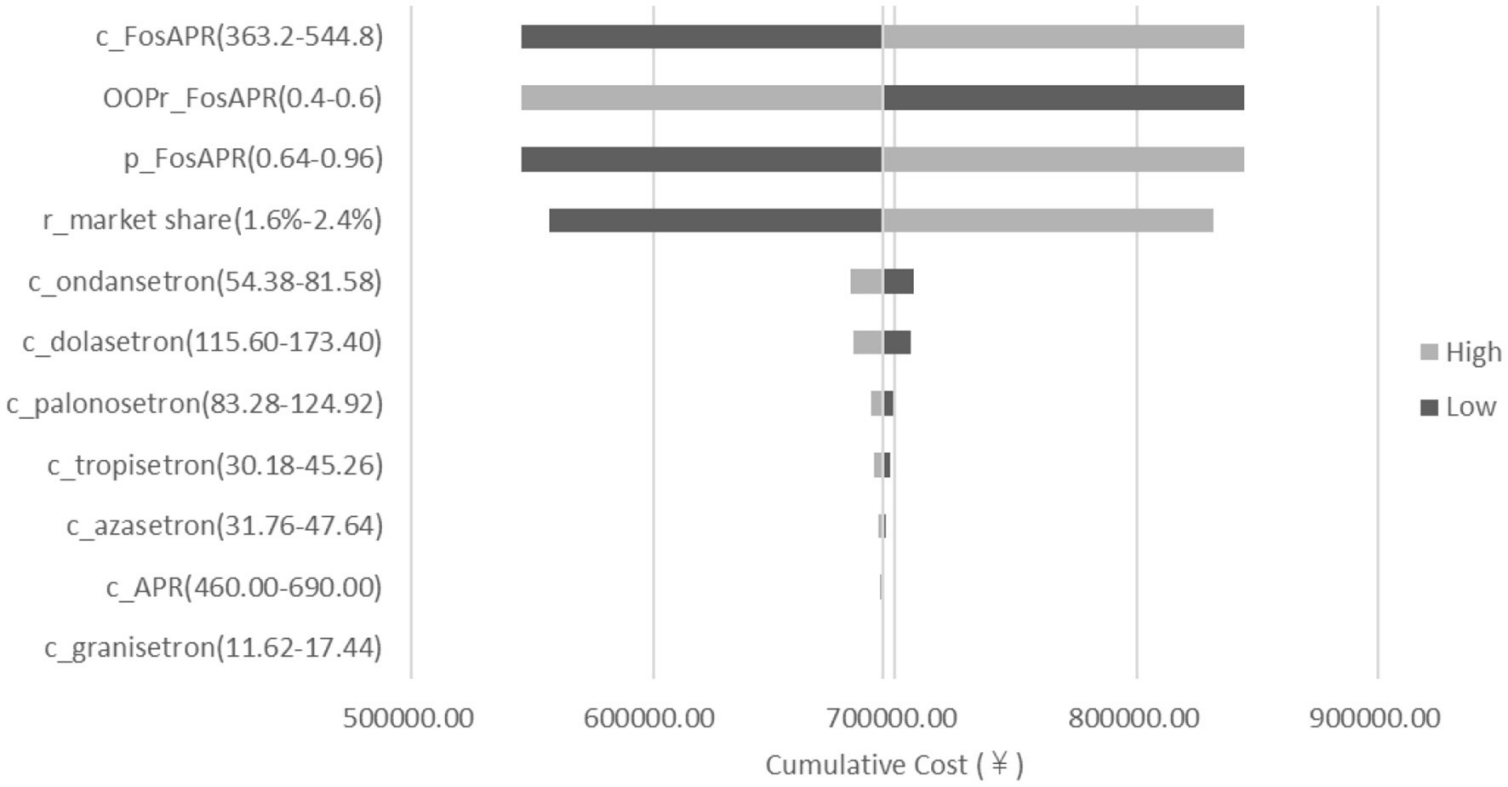
Deterministic sensitivity analysis tornado diagram of FosAPR in BIA. c, cost; p, proportion of reimbursement; OOPr, out-of-pocket ratio; r_market share, ratio of market share decline.

To test the robustness of the model, a deterministic sensitivity analysis was conducted on the drug prices, the ratio of market share decline, the health insurance OOP ratio, and the health insurance reimbursement rate of FosAPR. The range of each parameter was set between ±20%. The results of sensitivity analysis showed that the influence of the parameters on the results within the fluctuation of ±20% was similar to that of the base-case results, which meant the results of the BIA were stable. As shown in Figure 5, the price of FosAPR, the OOP ratio, and the proportion of reimbursement had a great impact on the results. Reducing the price or proportion of reimbursement, or increasing the OOP ratio of FosAPR could make the total payment decline significantly. The cumulative difference could reach ¥298,959 when the parameters fluctuated between ±20%. The high price of FosAPR might contribute to this result. On the premise of the OOP ratio and reimbursement ratio set in this study, if the drug price of FosAPR was < ¥32, it was possible to make the medical insurance payments equal to that in 2021. The ratio of market share decline had a certain influence on the result. When the ratio varied between 1.6 and 2.4%, the accumulated cost difference could reach ¥137,017 compared with the base-case result.

## Discussion

There were only a few published health technology assessment programs about FosAPR globally. To our knowledge, this is the first study to calculate the economic value of FosAPR for preventing CINV in patients who received moderate to high emetic chemotherapy from the perspective of the Chinese healthcare system for the affordability of health insurance funding. In detail, this study evaluated the cost-effectiveness of the FosAPR-containing regimen vs. the APR-containing regimen and conducted a budget impact analysis of the inclusion of FosAPR into the NRDL of China based on phase 3 clinical trial and real-world statistics.

In the cost-effectiveness analysis, although the APR-containing regimen had a higher cost than FosAPR, it gained a better outcome of QALY in patients. However, by calculating the ICER of two antiemetic drugs, we could not observe the pharmacoeconomic advantage of APR. As the cost of inpatient and other drugs in the antiemetic regimens were the same for both groups, the high drug price of APR might be the dominant factor that affected its economics. Despite the differences in the incidences of ADR, the disparities in administrations of rescue were minimal. The sensitivity analyses were conducted and confirmed the robustness of the model. With the expiration of the APR patent and the successive appearance on the market of its generic drugs, its cost-effectiveness might gradually emerge. Otherwise, if the price of APR remained high, for FosAPR, the probability of being more cost-effective in the acceptability curves would still be greater than that of APR in the Chinese background set by our study. In the European countries, Restelli et al. (34) constructed a Markov model to incorporate netupitant, aprepitant, and fosaprepitant into the cost-utility and budget impact analyses from the perspective of the Italian National Health Service (NHS). The results of the study illustrated that the netupitant-containing regimen was the most cost-effective. Besides, the cost-utility analysis conducted by Kashiwa and Matsushita (25) compared APR and FosAPR with a standard regimen based on data from two different trials, respectively, which revealed the cost-effectiveness of the addition of APR. Contrary to these studies, FosAPR showed an economic advantage in the context of the Chinese healthcare system. We speculated that it might be related to the following reasons. First, clinical data referred in studies were different in population and study design, which resulted in different response rates of patients. In Kashiwa's study, the economic results compared the addition of APR or FosAPR relatively with the standard regimen, forming an indirect comparison between APR and FosAPR. However, the clinical trial incorporated in this study was a randomized, parallel-group study in the Chinese population (27). Based on the trial, the conclusion of the cost-effectiveness of the FosAPR-containing regimen took the APR-containing regimen as the control group. Second, the health care program combined medical components in various combinations. There are huge differences in medical components between different countries, which led to cost differences. Third, from the perspective of Japanese payers, the price of FosAPR was higher than APR ($129.67 vs. 103.76). The total costs of FosAPR- or APR-containing regimen were $208.87 and 173.89, respectively, making the addition of FosAPR not cost-effective eventually. In China, the price negotiation between NHSA and drug manufacturers had significantly reduced the price of FosAPR (¥512.48 of FosAPR vs. ¥634.88 of APR), leading to its increase in cost-effectiveness.

As for the budget impact analysis, it might slightly increase the health insurance budget expenditure (1.49 million in 2021 to 1.84 million in 2024) and have a certain impact on the burden of the medical insurance fund if FosAPR was included in the NRDL of China. It could be related to the following factors. Firstly, the price of FosAPR was high, which was one of the most important reasons. The costs of other antiemetics were < ¥200 every single course. In addition, FosAPR needed to be combined with palonosetron for therapy, whose single course cost was more than ¥500. Therefore, the increase in market share after the addition of FosAPR in NRDL would inevitably lead to an increase in medical expenditure. However, the incremental budget was no more than 20% of the total amount of health insurance that covered antiemetic drugs every year. According to the Statistical Bulletin on the Development of Medical Security in 2020 (49), announced by the Nanjing Medical Security Bureau on 21 July 2021, the total expenditure of health insurance funds was ¥258,22 million. The cumulated incremental budget only accounted for a tiny proportion (0.003%) of the total expenditure, which may lead to a minor impact on the overall budget fund. Besides, the application of antiemetic drugs could reduce the mounting cost pressure on oncology to some extent. Secondly, the expected market share in 3 years of FosAPR in the model was much larger than those in actual status. According to the baseline proportion of drug purchases in Nanjing, the application of NK-1 RA in the market has not been widely promoted, so the promotion speed of NK-1 RA in the market was lower than our assumption. Therefore, the actual market share of FosAPR 3 years after its inclusion in the NRDL might be much smaller than the estimated share in the study. From the sensitivity analysis, we could see that if the ratio of market share decline dropped from 2 to 1.6%, the insurance budget and the cumulative costs would reduce accordingly compared with the base-case result. In other words, as the market promotion of FosAPR in real world could be smaller than our assumption, the actual impact on the insurance budget would be little after its inclusion in the NRDL. The results of BIA were based on the assumptions of the study and the estimates of the market, so the calculated health insurance expenditure of Nanjing might be overestimated, which was also one of the limitations of this BIA.

According to the guidelines and published pieces of literature (41–47), we found that chemotherapy was still one of the most effective and widely recommended methods for treatment in each period of cancer. Therefore, in this study, the incidence rate of five types of cancer in China was used to calculate the target population for receiving HEC. Similarly, in another Italian study that focused on the budget impact analysis of netupitant and palonosetron, the incidence rates of five types of cancer were also used to calculate the population for receiving HEC (34). However, with the development of new means of treatment, immunotherapy, targeted therapy, and genetic therapy were also used for some cancer patients with genetic mutations, making the proportion of patients receiving chemotherapy decrease. Therefore, the target population for receiving HEC was inevitably overestimated in the study. In general, the lack of rate of local HEC usage in China could lead to calculation errors in the target population, affecting the BIA results to some extent. In addition, due to the different medical insurance reimbursement policies across the country, this study only selected Nanjing urban medical insurance reimbursement policy as the reference for budget analysis, which might cause deviation in the results if the results were extended to the whole nation. This study also had some limitations in CEA. Firstly, we did not consider outpatients' situations because of the shortages in outpatients' research, which posed a certain obstacle to providing evidence for the medication chosen for outpatients. Secondly, we only brought direct medical costs in the CEA study, neglecting the indirect medical cost impacts on patients in the real world. In the real world, the hidden costs of chemotherapy were relatively exorbitant. Thirdly, the utility values of the three stages were not rigorous enough to reflect their true influence on QALYs.

The major strengths of this study are revealed in several ways. Firstly, our study filled the gap in the economic evaluation of antiemetic medicine. Secondly, it provided strong evidence for better drug choices for patients, and doctors, and for better planning of the NRDL.

## Conclusion

FosAPR had a non-inferior effect with APR and was cost-effective compared to APR at the current Chinese WTP threshold. The outcomes of the clinical trial and pharmacoeconomic evaluation both supported that FosAPR would be a better choice than APR to prevent CINV for patients who received emetic chemotherapy. In general, we could predict from the BIA results that the addition of FosAPR in the NRDL may mildly increase the burden of the public health insurance fund but also increase the coverage of patients who benefitted from FosAPR. The incremental BI of predicted years was relatively acceptable for the medical insurance fund. When considering whether the drug is included in the NRDL, the medical insurance payers should make a comprehensive investigation to negotiate the drug price, improve the economy of FosAPR, and the affordability of the medical insurance fund as much as possible.

## Data Availability Statement

The raw data supporting the conclusions of this article will be made available by the authors, without undue reservation.

## Ethics Statement

Ethical review and approval was not required for the study on human participants in accordance with the local legislation and institutional requirements. Written informed consent from the patients/participants or patients/participants' legal guardian/next of kin was not required to participate in this study in accordance with the national legislation and the institutional requirements.

## Author Contributions

NZ and XL designed the whole study. XX, YB, KX, and ZZ were responsible for collecting and analyzing data. XX and ZZ conducted the models of the study. XX and YB contributed to the original draft of the study. ZZ, NZ, and XL took the responsibility for review and editing. All authors contributed to the article and approved the submitted version.

## Funding

This work was supported by the National Natural Science Foundation of China (72074123 and 71673147) and the China Medical Board (Grant No: 17-277).

## Conflict of Interest

The authors declare that the research was conducted in the absence of any commercial or financial relationships that could be construed as a potential conflict of interest.

## Publisher's Note

All claims expressed in this article are solely those of the authors and do not necessarily represent those of their affiliated organizations, or those of the publisher, the editors and the reviewers. Any product that may be evaluated in this article, or claim that may be made by its manufacturer, is not guaranteed or endorsed by the publisher.

## References

[1] Bray F Ferlay J Soerjomataram I Siegel RL Torre LA Jemal A. Global cancer statistics 2018: GLOBOCAN estimates of incidence and mortality worldwide for 36 cancers in 185 countries. CA Cancer J Clin. (2018) 68:394–424. 10.3322/caac.21492 30207593

[2] Sung H Ferlay J Siegel RL Laversanne M Soerjomataram I Jemal A. Global cancer statistics 2020: GLOBOCAN estimates of incidence and mortality worldwide for 36 cancers in 185 countries. CA Cancer J Clin. (2021) 71:209–49. 10.3322/caac.21660 33538338

[3] Perwitasari DA Atthobari J Mustofa M Dwiprahasto I Hakimi M Gelderblom H. impact of chemotherapy-induced nausea and vomiting on quality of life in Indonesian patients with gynecologic cancer. Int J Gynecol Cancer. (2012) 22:139–45. 10.1097/IGC.0b013e318234f9ee 22080888

[4] National Comprehensive Cancer Network (NCCN). NCCN Clinical Practice Guidelines in Oncology: Antiemesis Version 2.2019. Bethesda, MD (2019).

[5] The Committee of Rehabilitation and Palliative Care (Shanghai). Shanghai expert consensus on the management of chemotherapy-induced nausea and vomiting (Version 2018). China Oncol. (2018) 28:946–60 (in Chinese) 10.19401/j.cnki.1007-3639.2018.12.011

[6] Zhang B Li XL Adherence to clinical guidelines for prophylaxis of chemotherapy induced nausea and vomiting. Chin Hosp Pharm J. (2018) 38:1325–9 (in Chinese). 10.13286/j.cnki.chinhosppharmacyj.2018.12.19

[7] Hesketh PJ Bohlke K Lyman GH Basch E Chesney M Clark-Snow RA. Antiemetics: American Society of Clinical Oncology Focused Guideline Update. J Clin Oncol. (2016) 34:381–6. 10.1200/JCO.2015.64.3635 26527784

[8] Warr DG Gr/unberg SM Gralla RJ Hesketh PJ Roila F Wit Rd. The oral NK1 antagonist aprepitant for the prevention of acute and delayed chemotherapy-induced nausea and vomiting: pooled data from 2 randomised, double-blind, placebo controlled trials. Eur J Cancer. (2005) 41:1278–85. 10.1016/j.ejca.2005.01.024 15939263

[9] Tsuji D Suzuki K Kawasaki Y Goto K Matsui R Seki N. Risk factors associated with chemotherapy-induced nausea and vomiting in the triplet antiemetic regimen including palonosetron or granisetron for cisplatin-based chemotherapy: analysis of a randomized, double-blind controlled trial. Support Care Cancer. (2019) 27:1139–47. 10.1007/s00520-018-4403-y 30094732

[10] Iihara H Fujii H Yoshimi C Yamada M Suzuki A Matsuhashi N. Control of chemotherapy-induced nausea in patients receiving outpatient cancer chemotherapy. Int J Clin Oncol. (2016) 21:409–18. 10.1007/s10147-015-0908-2 26475354 PMC4824820

[11] Lee KM Jung DY Hwang H Kim WH Lee JY Kim TY. Late chronotypes are associated with neoadjuvant chemotherapy-induced nausea and vomiting in women with breast cancer. Chronobiol Int. (2017) 34:480–91. 10.1080/07420528.2017.1295978 28362229

[12] Mosa ASM Hossain AM Lavoie BJ Yoo I. Patient-related risk factors for chemotherapy-induced nausea and vomiting: a systematic review. Front Pharmacol. (2020) 11:329. 10.3389/fphar.2020.00329 32296333 PMC7138899

[13] Yu SY Yin JL Qin SK Wang JJ Chen Y Shen L. Guidelines for the prevention and treatment of vomiting associated with tumor therapies (Version 2014). Chin Clin Oncol. (2014) 19:263–73 (in Chinese).

[14] Kang HJ Loftus S Taylor A DiCristina C Green S Zwaan CM. Aprepitant for the prevention of chemotherapy-induced nausea and vomiting in children: a randomised, double-blind, phase 3 trial. Lancet Oncol. (2015) 16:385–94. 10.1016/S1470-2045(15)70061-6 25770814

[15] Aapro MS Schmoll HJ Jahn F Carides AD Webb RT. Review of the efficacy of aprepitant for the prevention of chemotherapy-induced nausea and vomiting in a range of tumor types. Cancer Treat Rev. (2013) 39:113–7. 10.1016/j.ctrv.2012.09.002 23062719

[16] Xiong J Zhao G Yang S Chen J. Efficacy, tolerability and pharmacokinetic impact of aprepitant in sarcoma patients receiving ifosfamide and doxorubicin chemotherapy: a randomized controlled trial. Adv Ther. (2019) 36:355–64. 10.1007/s12325-018-0862-2 30607545

[17] Colon-Gonzalez F Kraft WK. Pharmacokinetic evaluation of fosaprepitant dimeglumine. Expert Opin Drug Metab Toxicol. (2010) 6:1277–86. 10.1517/17425255.2010.513970 20795794 PMC3155701

[18] Jordan K Gralla R Jahn F Molassiotis A. International antiemetic guidelines on chemotherapy induced nausea and vomiting (CINV): content and implementation in daily routine practice. Eur J Pharmacol. (2014) 722:197–202. 10.1016/j.ejphar.2013.09.073 24157984

[19] Moore S Tumeh J Wojtanowski S Flowers C. Cost-effectiveness of aprepitant for the prevention of chemotherapy-induced nausea and vomiting associated with highly emetogenic chemotherapy. Value Health. (2007) 10:23–31. 10.1111/j.1524-4733.2006.00141.x 17261113

[20] Humphreys S Pellissier J Jones A. Cost-effectiveness of an aprepitant regimen for prevention of chemotherapy-induced nausea and vomiting in patients with breast cancer in the UK. Cancer Manag Res. (2013) 5:215–24. 10.2147/CMAR.S44539 23950658 PMC3742066

[21] Annemans L Strens D Lox E Petit C Malonne H. Cost-effectiveness analysis of aprepitant in the prevention of chemotherapy-induced nausea and vomiting in Belgium. Support Care Cancer. (2008) 16:905–15. 10.1007/s00520-007-0349-1 17965891

[22] Lordick F Ehlken B Ihbe-Heffinger A Berger K Krobot KJ Pellissier J. Health outcomes and cost-effectiveness of aprepitant in outpatients receiving antiemetic prophylaxis for highly emetogenic chemotherapy in Germany. Eur J Cancer. (2007) 43:299–307. 10.1016/j.ejca.2006.09.019 17134890

[23] Chan SL Jen J Burke T Pellissier J. Economic analysis of aprepitant-containing regimen to prevent chemotherapy-induced nausea and vomiting in patients receiving highly emetogenic chemotherapy in Hong Kong. Asia Pac J Clin Oncol. (2014) 10:80–91. 10.1111/ajco.12170 24571059

[24] Lopes G Burke T Pellissier J Zhang XH Dedhiya S Chan A. Aprepitant for patients receiving highly emetogenic chemotherapy: an economic analysis for Singapore. Value Health Reg Issues. (2012) 1:66–74. 10.1016/j.vhri.2012.03.002 29702829

[25] Kashiwa M Matsushita R. Comparative cost-utility analysis between aprepitant- and fosaprepitant-containing regimens to prevent chemotherapy-induced nausea and vomiting in patients receiving highly emetogenic chemotherapy in Japan. Clin Ther. (2019) 41:929–42. 10.1016/j.clinthera.2019.03.011 31036286

[26] Pearson SD. The ICER value framework: integrating cost effectiveness and affordability in the assessment of health care value. Value Health. (2018) 21:258–65. 10.1016/j.jval.2017.12.017 29566831

[27] Yang LQ Sun XC Qin SK Cheng Y Shi JH Chen ZD. Efficacy and safety of fosaprepitant in the prevention of nausea and vomiting following highly emetogenic chemotherapy in Chinese people: A randomized, double-blind, phase III study. Eur J Cancer Care. (2017) 26:e12668. 10.1111/ecc.12668 28393417 PMC5697660

[28] Sun CC Bodurka DC Weaver CB Rasu R Wolf JK Bevers MW. Rankings and symptom assessments of side effects from chemotherapy: insights from experienced patients with ovarian cancer. Support Care Cancer. (2005) 13:219–27. 10.1007/s00520-004-0710-6 15538640

[29] Kashiwa M Matsushita R. Cost-utility analysis of palonosetron in the antiemetic regimen for cisplatin-containing highly emetogenic chemotherapy in Japan. BMC Health Serv Res. (2019) 19:438. 10.1186/s12913-019-4281-0 31262292 PMC6604132

[30] Mauskopf JA Sullivan SD Annemans L Caro J Mullins CD Nuijten M. Principles of good practice for budget impact analysis: report of the ISPOR Task Force on good research practices–budget impact analysis. Value Health. (2007) 10:336–47. 10.1111/j.1524-4733.2007.00187.x 17888098

[31] Marshall DA Douglas PR Drummond MF Torrance GW Macleod S Manti O. Guidelines for conducting pharmaceutical budget impact analyses for submission to public drug plans in Canada. Pharmacoeconomics. (2008) 26:477–95. 10.2165/00019053-200826060-00003 18489199

[32] Nanjing Municipal Bureau Statistics. Nanjing Statistical Yearbook 2020. Available online at: http://tjj.nanjing.gov.cn/material/njnj_2020/renkou/3-1.htm (accessed April 05, 2022).

[33] Chen W Zheng R Baade PD Zhang S Zeng H Bray F. Cancer statistics in China, 2015. CA Cancer J Clin. (2016) 66:115–32. 10.3322/caac.21338 26808342

[34] Restelli U Saibene G Nardulli P Di Turi R Bonizzoni E Scolari F. Cost-utility and budget impact analyses of the use of NEPA for chemotherapy-induced nausea and vomiting prophylaxis in Italy. BMJ Open. (2017) 7:e015645. 10.1136/bmjopen-2016-015645 28765126 PMC5642784

[35] Feng RM Zong YN Cao SM Xu RH. Current cancer situation in China: good or bad news from the 2018 global cancer statistics? Cancer Commun. (2019) 39:22. 10.1186/s40880-019-0368-6 31030667 PMC6487510

[36] Argirion I Zarins KR Defever K Suwanrungruang K Chang JT Pongnikorn D. Temporal changes in head and neck cancer incidence in Thailand suggest changing oropharyngeal epidemiology in the region. J Glob Oncol. (2019) 5:1–11. 10.1200/JGO.18.00219 30860955 PMC6449079

[37] Sadeghi M Ghoncheh M Mohammadian-Hafshejani A Gandomani HS Rafiemanesh H Salehiniya H. Incidence and mortality of testicular cancer and relationships with development in Asia. Asian Pac J Cancer Prev. (2016) 17:4251–7. 27797227

[38] Fu J Wu B. Budget impact analysis of afatinib in advanced non-small cell lung cancer patients with EGFR mutation. Chin J Mod Appl Pharm. (2019) 36:723–5 (in Chinese). 10.13748/j.cnki.issn1007-7693.2019.06.016

[39] Sullivan SD Mauskopf JA Augustovski F Jaime Caro J Lee KM Minchin M. Budget impact analysis-principles of good practice: report of the ISPOR 2012 Budget Impact Analysis Good Practice II Task Force. Value Health. (2014) 17:5–14. 10.1016/j.jval.2013.08.2291 24438712

[40] Chinese Society of Clinical Oncology. Guidelines of Chinese Society of Clinical Oncology (CSCO) Prevention & Treatment of Nausea and Vomiting Caused by Antitumor Therapies (Version 2019). Beijing: People's Medical Publishing House (PMPH) (2019). 10.13699/j.cnki.1001-6821.2015.03.008

[41] Chinese Medical Association. Guidelines for clinical diagnosis and treatment of lung cancer of Chinese Medical Association (Version 2019). Chin J Oncol. (2020) 42:257–87 (in Chinese). 10.3760/cma.j.cn112152-20200120-00049 32375445

[42] National Comprehensive Cancer Network (NCCN). NCCN Clinical Practice Guidelines in Oncology-Gastric Cancer (Version 3.2020). Available online at: http://www.nccn.org/ (accessed April 05, 2022).

[43] Kaushal P Atri R Soni A Kaushal V. Comparative evaluation of triplet antiemetic schedule versus doublet antiemetic schedule in chemotherapy-induced emesis in head and neck cancer patients. Ecancermedicalscience. (2015) 9:567. 10.3332/ecancer.2015.567 26435740 PMC4583242

[44] Bhide SA Nutting CM. Advances in chemotherapy for head and neck cancer. Oral Oncol. (2010) 46:436–8. 10.1016/j.oraloncology.2010.03.004 20400360

[45] Lorch A Albers P. Update Erstlinien- und Rezidivchemotherapie beim Hodentumor [Update on first-line and relapse chemotherapy for testicular cancer]. Urologe A. (2013) 52:1547–55. 10.1007/s00120-013-3251-0 24126502

[46] Kamat AM Hahn NM Efstathiou JA Lerner SP Malmström PU Choi W. Bladder cancer. Lancet. (2016) 388:2796–810. 10.1016/S0140-6736(16)30512-8 27345655

[47] Luo ST Wang YH Liao DZ Wang MJ Li XK Tang W. Efficacy and safety of pirarubicin hot perfusion combined with systemic chemotherapy in the treatment of advanced bladder cancer. Chin J Clin Pharmacol. (2015) 31:184–6 (in Chinese). 10.13699/j.cnki.1001-6821.2015.03.008

[48] Medical Security Bureau, Nanjing. Urban Medical Insurance Drug List Query. Available online at: http://ybj.nanjing.gov.cn/ (accessed April 05, 2022).

[49] Nanjing Medical Security Bureau. Medical Security Development Statistics Nanjing 2020 (2021). Available from: http://ybj.nanjing.gov.cn/gkml/202107/t20210702_3024626.html (accessed April 05, 2022).

